# Supplementary material for: Heatstroke-induced hepatocyte exosomes promote liver injury by activating the NOD-like receptor signaling pathway in mice
Source: PeerJ. 2019 Dec 19;7:e8216. doi: 10.7717/peerj.8216 (PMC6925953; doi:10.7717/peerj.8216)
Supplement: File S20 [file peerj-07-8216-s024.docx]

**Table S1.** The related parameters used in identification and quantitative analyses by mass spectrometry

| **Item** | **Value** |
| --- | --- |
| Protein Database | Uniprot_HomoSapiens_161584_20180123  Download link：<http://www.uniprot.org/> |
| Enzyme | Trypsin |
| Max Missed Cleavages | 2 |
| Instrument | ESI-TRAP |
| Precursor Mass Tolerance | ± 20 ppm |
| Fragment Mass Tolerance | 0·1 Da |
| Use Average Precursor Mass | False |
| Modification Groups from Quan Method | iTRAQ 4 plex |
| Dynamic modifications | Oxidation (M), Acetyl (Protein N-term), Deamidated (NQ) |
| Static modifications | Carbamidomethyl (C) |
| Database pattern | Decoy |
| Peptide FDR | ≤0.01 |
